# Identifying Inequities in Video and Audio Telehealth Services for Primary Care Encounters During COVID-19: Repeated Cross-Sectional, Observational Study

**DOI:** 10.2196/49804

**Published:** 2023-09-29

**Authors:** Lorraine R Buis, Lindsay K Brown, Melissa A Plegue, Reema Kadri, Anna R Laurie, Timothy C Guetterman, V G Vinod Vydiswaran, Jiazhao Li, Tiffany C Veinot

**Affiliations:** 1 Department of Family Medicine University of Michigan Ann Arbor, MI United States; 2 School of Information University of Michigan Ann Arbor, MI United States; 3 Department of Pediatrics University of Michigan Ann Arbor, MI United States; 4 Department of Learning Health Sciences University of Michigan Medical School Ann Arbor, MI United States; 5 Department of Health Behavior and Health Education School of Public Health University of Michigan Ann Arbor, MI United States

**Keywords:** COVID-19, telemedicine, health equity, clinical encounters, electronic health records

## Abstract

**Background:**

The COVID-19 pandemic resulted in rapid changes in how patient care was provided, particularly through the expansion of telehealth and audio-only phone-based care.

**Objective:**

The goal of this study was to evaluate inequities in video and audio-only care during various time points including the initial wave of the COVID-19 pandemic, later stages of the pandemic, and a historical control. We sought to understand the characteristics of care during this time for a variety of different groups of patients that may experience health care inequities.

**Methods:**

We conducted a retrospective analysis of electronic health record (EHR) data from encounters from 34 family medicine and internal medicine primary care clinics in a large, Midwestern health system, using a repeated cross-sectional, observational study design. These data included patient demographic data, as well as encounter, diagnosis, and procedure records. Data were obtained for all in-person and telehealth encounters (including audio-only phone-based care) that occurred during 3 separate time periods: an initial COVID-19 period (T2: March 16, 2020, to May 3, 2020), a later COVID-19 period (T3: May 4, 2020, to September 30, 2020), and a historical control period from the previous year (T1: March 16, 2019, to September 30, 2019). Primary analysis focused on the status of each encounter in terms of whether it was completed as scheduled, it was canceled, or the patient missed the appointment. A secondary analysis was performed to evaluate the likelihood of an encounter being completed based on visit modality (phone, video, in-person).

**Results:**

In total, there were 938,040 scheduled encounters during the 3 time periods, with 178,747 unique patients, that were included for analysis. Patients with completed encounters were more likely to be younger than 65 years old (71.8%-74.1%), be female (58.8%-61.8%), be White (75.6%-76.7%), and have no significant comorbidities (63.2%-66.8%) or disabilities (53.2%-61.1%) in all time periods than those who had only canceled or missed encounters. Effects on different subpopulations are discussed herein.

**Conclusions:**

Findings from this study demonstrate that primary care utilization across delivery modalities (in person, video, and phone) was not equivalent across all groups before and during the COVID-19 pandemic and different groups were differentially impacted at different points. Understanding how different groups of patients responded to these rapid changes and how health care inequities may have been affected is an important step in better understanding implementation strategies for digital solutions in the future.

## Introduction

The COVID-19 pandemic disrupted nearly every facet of life across the globe. In the United States, the impact on the health care system has been enormous and has resulted in rapid change in how patient care is provided—particularly through the expansion of telehealth and audio-only phone-based care. Despite decades of promise for telehealth, illustrated by many demonstration projects documenting feasibility, acceptability, and efficacy of delivering care remotely [1], large-scale adoption prior to COVID-19 was slow. This was due to barriers related to cost and reimbursement, licensure and practicing across state lines, and patient and provider comfort [2-4]. Moreover, prior to COVID-19, audio-only phone-based care was rarely considered within the scope of telehealth services and was not routinely reimbursed by public or private payers. Temporary changes enacted by the US Centers for Medicare & Medicaid Services (CMS) during the pandemic changed this, opening the door to increased telehealth utilization. For many health systems, COVID-19 was the catalyst that truly brought telehealth into routine clinical practice.

Although many successes with telehealth adoption have been documented during the COVID-19 pandemic, concerns regarding the potential for adverse impacts on existing health care inequities have been raised [5]. Populations such as historically marginalized racial and ethnic groups, older adults, groups experiencing socioeconomic marginalization, and those shouldering the burdens of adverse social determinants of health have historically suffered from health and health care inequities [6] and also experienced greater burdens from COVID-19, including morbidity and mortality [7-11]. These same groups also have lower rates of home broadband and smartphone adoption [12], as well as lower rates of patient portal adoption [13], which may directly impact access to virtual care. Moreover, research documenting telehealth utilization often tells an incomplete story and does not necessarily document the extent to which inequities in care during the COVID-19 pandemic may have been realized. Recent work has focused primarily on documenting differences in completed clinical encounters, which often ignores encounters that were canceled prior to the scheduled appointment time or encounters where patients did not attend their scheduled appointment. Although canceled encounters and missed encounters have always been a problem in the health care system, COVID-19 and the expanded use of telehealth had the potential to exacerbate these issues for various groups for which technology literacy and access issues may have influenced the ability for patients to seek care. Focusing attention on these additional scenarios is an important part of understanding access to care during the COVID-19 pandemic.

Of particular concern regarding access to care is the fact that previous health informatics interventions have often increased intervention-generated inequalities, as people who have more socioeconomic advantages tend to have increased access, uptake, and adherence to these interventions relative to people who are more economically marginalized [14]. Initial evidence suggests that this trend may hold for video and audio-only care. Indeed, recent work has identified inequities in telehealth use at health systems during the early days of the COVID-19 pandemic, with specific focus on older adults as well as historically marginalized racial and ethnic groups [5]. Despite this evidence for increased health care inequity in the early stages of the COVID-19 pandemic concerning video and audio-only care, few studies have looked beyond completed encounters to tell a more robust and complete story of how care and access to care changed during the pandemic, as well as the effect that change may have had on different groups of patients.

The goal of this study was to evaluate inequities in video and audio-only care during various time points including the initial wave of the COVID-19 pandemic, later stages of the pandemic, and a historical control. We sought to understand the characteristics of care during this time for a variety of different groups of patients who may experience health care inequity. In contrast to previously published work that has mostly focused on encounter modality without a historical control, we focused also on encounters that were completed as well as encounters that were canceled or where the patient did not attend (discussed here as “missed” encounters). Looking at data from our different time points, focusing on different care delivery modalities, and paying attention to different encounter statuses (completed, canceled, or missed) allowed us to understand the effects of COVID-19 on telehealth implementation and health care access inequities more robustly.

## Methods

**Study Design, Setting, and Data Source**

To evaluate provision of care during the COVID-19 pandemic, we conducted a repeated cross-sectional, observational study of electronic health record (EHR) data obtained from encounters from 34 family medicine and internal medicine primary care clinics in a large, Midwestern health system. These data include patient demographic data, as well as encounter, diagnosis, and procedure records. Data were obtained for all in-person and telehealth encounters (including audio-only phone-based care) that occurred during 3 separate time periods: an initial COVID-19 period (T2: March 16, 2020, to May 3, 2020), a later COVID-19 period (T3: May 4, 2020, to September 30, 2020), and a historical control period from the previous year (T1: March 16, 2019, to September 30, 2019). The historical control (T1) was further subdivided into T1a and T1b for specific analyses so that matched comparisons with T2 and T3 could be made. The dates of the T2 and T3 COVID-19 time periods were chosen purposefully, as T2 coincided with the strategic ramp down of ambulatory care encounters at the health system in favor of virtual care modalities. This ramp down occurred as part of an effort to mitigate the risk of patient and staff exposure to COVID-19 when possible. In contrast, T3 marked the health system ramp up period in which patients were brought back into the clinic.

**Procedures**

To ensure data were comparable across time periods and to focus on health care provision for traditionally scheduled encounters with a provider, data were reduced in several ways. First, COVID-19 testing encounters during the pandemic period were removed from the data set. As the focus of the research was on visits for which telehealth was possible, duplicated encounter records and data for completed (ie, no record of cancellation or missed) encounters without associated clinical notes, including visits for immunizations, lab work, radiology scans, and procedures, were removed. Telephone encounters were filtered to include only scheduled health care visits by restricting to those that had associated scheduled appointment times, record of cancellation or missed status, billing or visit diagnosis, or procedure codes. This filtering of telephone visits was intended to remove phone calls that were for administrative or scheduling purposes, as well as calls for nonurgent health questions. Finally, as children have a unique care trajectory as compared with adults (ie, they are not scheduling their own appointments or managing the logistics of receiving virtual or in-person care on their own), we limited analyses to patients 18 years old or older. All data cleaning and analyses were conducted using R statistical software [15]. The STROBE (Strengthening the Reporting of Observational Studies in Epidemiology) checklist for reporting cross-sectional, observational studies was utilized to ensure robust reporting in this paper [16].

**Primary Analysis**

Primary analysis focused on the status of each encounter, in terms of whether it was completed as scheduled, it was canceled, or the patient missed the encounter. Completed encounters were identified by having both check-in and checkout times and associated billing activity. Canceled encounters were identified by cancellation times or reasons listed in the patient’s chart. Finally, missed encounters were identified as those encounters that either (1) listed missed encounter activity (ie, letter sent to patient about a missed appointment) or (2) did not have check-in and checkout times or associated billing activity that were also not listed as being canceled. To aid in interpretation, a separate binary logistic regression model was run for each of the 3 possible outcome statuses (completed, canceled, or missed). Analysis was conducted at the encounter level utilizing a generalized estimating equation (GEE) framework to account for correlations present due to multiple encounters for any given patient. Patient-level covariates included in these models were race, ethnicity, age (dichotomized to less than 65 years and 65 years or older), sex (male or female), and presence of comorbidities. Comorbidities and disabilities were identified using ICD (International Classification of Diseases) codes from problem summary lists and visit diagnoses based on the time of a given encounter. Comorbidity scores were calculated as weighted Charlson indexes [17] using R’s comorbidity package [18]. These scores were subsequently converted to a categorical variable with 2 values: 0 significant comorbidities or ≥1 significant comorbidity. Disability status was identified by a recorded diagnosis of any condition associated with cognitive, mobility, vision, or hearing disability. Some conditions that cause disability may have also been included in the Charlson Comorbidity Index (ie, diabetes-related mobility or vision issues), but the overall categorization of the 2 groups had different functions in our analysis. Comorbidity was meant to establish the likelihood of a patient requiring health care, and disability represented a potential barrier to accessing health care. All covariates were interacted with the time period of the encounter in order to assess the differences in the impact of the various patient characteristics across the 3 time periods.

**Secondary Analysis**

A secondary analysis was performed to evaluate the likelihood of an encounter being completed based on visit modality (phone, video, in-person). A model analogous to those run for overall encounter status was run focusing only on whether the outcome was completed (vs canceled or missed). Covariates for this model were the same as in the primary models; however, instead of interacting by time period, time period was included as a covariate, and all covariates were interacted by visit modality. Data were limited to the COVID-19 time periods only (T2 and T3), as telehealth was not routinely used during the historical control period and there were only 40 video encounters during that time.

**Ethics Statement**

This study was determined to be exempt from review by the University of Michigan Human Subjects Review Board (HUM00187621).

## Results

### Encounter Characteristics

In total, there were 938,040 scheduled encounters during the 3 time periods, with 178,747 unique patients, that were included for analysis (Table 1). During the historical control period (2019), scheduled encounters included 40 (38 completed) video visits, 181,864 (180,222 completed) phone visits, and 282,496 (186,109 completed) in-person visits. During the subsequent pandemic year (2020, including both T2 and T3), video visits sharply increased in number to 70,170 scheduled encounters (66,550 completed), phone visits slightly increased to 202,513 scheduled encounters (200,348 completed), and in-person encounters decreased to 200,957 scheduled encounters (81,833 completed). Patients with completed encounters were more likely to be younger than 65 years (T1: 89,451/120,943, 74%; T2: 25,659/35,724, 71.8%; T3: 79,115/106,708, 74.1%), be female (T1: 71,133/120,943, 58.8%; T2: 22,061/35,724, 61.8%; T3: 63,163/106,708, 59.2%), be White (T1: 92,728/120,943, 76.7%; T2: 27,014/35,724, 75.6%; T3: 80,636/106,708, 75.6%), and have no significant comorbidities (T1: 80,802/120,943, 66.8%; T2: 20,843/35,724, 58.3%; T3: 67,472/106,708, 63.2%) nor disabilities (T1: 73,937/120,943, 61.1%; T2: 18,994/35,724, 53.2%; T3: 64,420/106,708, 58.5%) in all time periods than those who had only canceled or missed encounters (Table 1). Relative demographic percentages were consistent across all 3 time periods, except for slight differences at T2, when COVID-19 shutdowns were in full effect. At time T2, patients who were at least 65 years old; were female; were Black; had at least one significant comorbidity; or had at least one mobility, cognition, vision, or hearing-related disability were represented in a slightly higher percentage of completed encounters, as compared with these same groups at the other 2 time periods. The opposite was true for Asian patients, who were represented in a slightly smaller percentage of completed encounters, as compared with these patients at T1 and T3. Patient insurance was only provided at the encounter level for completed encounters (with associated billing) and therefore could not be assessed adequately in our encounter-level analysis for canceled and missed encounters. However, most completed and billed encounters were paid via commercial insurance (T1: 89,723/120,943, 74.2%; T2: 24,959/35,724, 69.8%; T3: 73,608/106,708, 68.9%) at all time periods, with Medicare being used in approximately 20% (T1: 20,399/120,943, 16.9%; T2: 6794/35,724, 19%; T3: 17,748/106,708, 15.9%) of these encounters. Please see Table 2 for complete details on the demographics of patients with scheduled visits by time period and visit status.

Encounter status, whether an encounter was completed, canceled, or missed, was assessed across the study time periods for each visit type included in the study (in-person, phone, or video; Table 3). When viewing T1 as 2 separate time periods to correspond with the COVID-19 era time periods, T1a (March 16, 2019, to May 3, 2019) and T1b (May 4, 2019, to September 30, 2019) to match the time frames for T2 and T3, respectively, we observed a marked drop in completed in-person encounters at both T2 and T3 as compared with the corresponding T1 timeframe, as well as a slight uptick in encounters completed by phone between T1b and T3 (Table 3). Canceled in-person appointments increased at both T2 and T3 compared with their respective timeframes within T1; however, missed appointments decreased for both time periods. The status breakdown for phone encounters was similar at T1b and T3, with significant drops in appointments of all statuses when comparing T1a with T2. Conversely but unsurprisingly, total scheduled video encounters went from very few encounters throughout our historical control T1 (n=40) to becoming much more common at T2 (n=17,233) and T3 (n=52,937) across all encounter status types.

**Figure 1:**
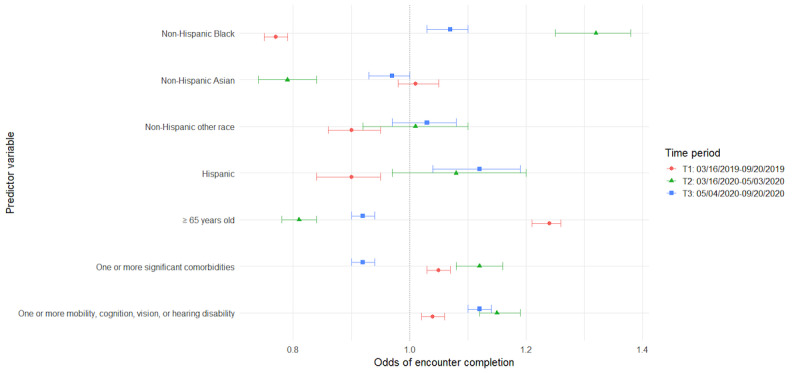
Predictors of encounter completion by time period.

**Figure 2:**
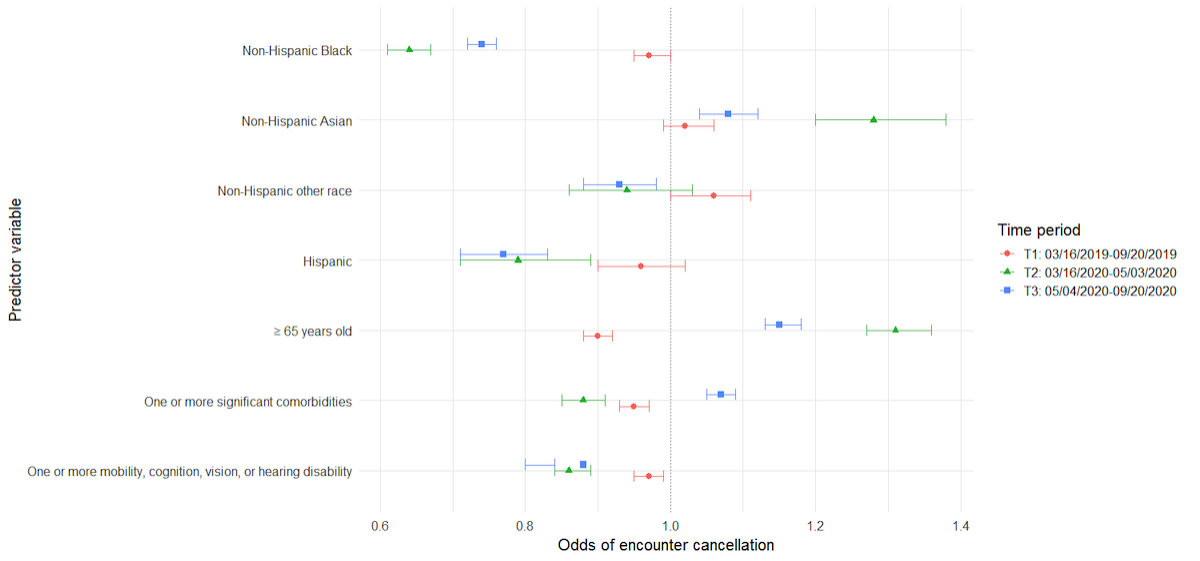
Predictors of encounter cancellation by time period.

**Figure 3:**
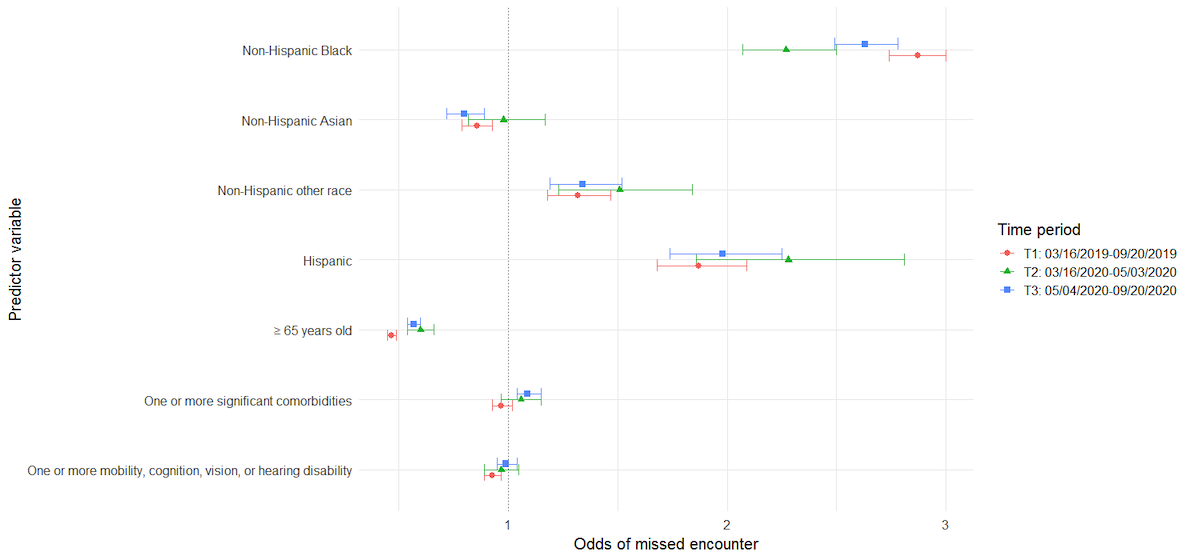
Predictors of missed encounters by time period.

**Figure 4:**
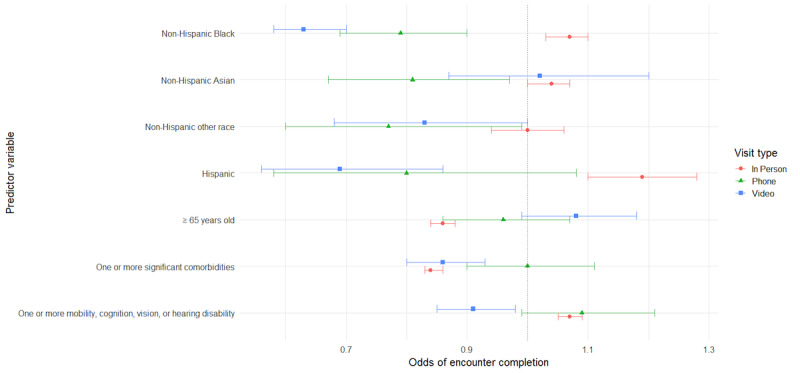
Predictors of encounter completion by visit type.

The following sections detail the results from a series of GEE models for the primary and secondary analyses detailed in the methods (Tables 4-7). For ease of interpretation, results are presented by patient characteristics. Additionally, visual representations of odds ratios (ORs) across time for each of the models are provided to aid in interpretation (Figures 1-4). The vertical dotted line in each figure indicates an OR of 1 (no difference in odds). Error bars to the right of this line indicate increased odds for the group indicated, error bars to the left of this line indicate reduced odds, and error bars intersecting this line indicate no significant difference in odds between the indicated group and reference group.

**Table 1:** Number and percent of all scheduled encounters (n=938,040) by encounter status and visit modality.

Visit modality	Completed (n=715,100), n (%)	Canceled (n=192,646), n (%)	Missed (n=30,294), n (%)	Total sample, n (%)
Video	66,588 (9.31)	1526 (0.79)	2096 (6.92)	70,210 (7.48)
Phone	380,570 (53.22)	94 (0)	3713 (12.26)	384,377 (40.98)
In-person	267,942 (37.47)	191,026 (99.16)	24,485 (80.82)	483,453 (51.54)

**Table 2:** Demographics of patients with any scheduled visit by time period and visit status.

Characteristic			T1 (March 16, 2019, to September 20, 2019), n (%)					T2 (March 16, 2020, to May 3, 2020), n (%)					T3 (May 4, 2020, to September 20, 2020), n (%)		
			No completed encounters (n=10,393)		At least 1 completed encounter (n=120,943)		No completed encounters (n=16,807)			At least 1 completed encounter (n=35,724)		No completed encounters (n=12,568)			At least 1 completed encounter (n=106,708)
**Race/ethnicity**															
	Non-Hispanic White	7394 (71.1)		92,728 (76.7)		12,857 (76.5)			27,014 (75.6)		9428 (75.0)			80,636 (75.6)	
	Non-Hispanic Black	1259 (12.1)		11,255 (9.3)		1407 (8.4)			4518 (12.6)		1163 (9.3)			10,028 (9.4)	
	Non-Hispanic Asian	968 (9.3)		10874 (9.0)		1637 (9.7)			2311 (6.5)		1240 (9.9)			10,038 (9.4)	
	Non-Hispanic other race	421 (4.1)		3750 (3.1)		546 (3.2)			1093 (3.1)		366 (2.9)			3509 (3.3)	
	Hispanic	229 (2.2)		2242 (1.9)		296 (1.8)			721 (2.0)		237 (1.9)			1968 (1.8)	
	Missing	122 (1.2)		94 (0.1)		64 (0.4)			67 (0.2)		134 (1.1)			529 (0.5)	
**Age at encounter (years)**															
	18-64	8751 (84.2)		89,451 (74)		12,124 (72.1)			25,659 (71.8)		9758 (77.6)			79,115 (74.1)	
	≥65	1642 (15.8)		31,492 (26)		4683 (27.9)			10,065 (28.2)		2810 (22.4)			27,593 (25.9)	
**Weighted Charlson Index**															
	Zero significant comorbidities	2710 (26.1)		80,802 (66.8)		9179 (54.6)			20,843 (58.3)		5932 (47.2)			67,472 (63.2)	
	One or more significant comorbidities	708 (6.8)		33,841 (27.9)		4462 (26.6)			12,578 (35.2)		2180 (17.3)			29,913 (28)	
	Missing	6975 (67.1)		6300 (5.2)		3166 (18.8)			2303 (6.4)		4456 (35.5)			9323 (8.7)	
**Disability status**															
	No identified disabilities	4632 (44.6)		73,937 (61.1)		9701 (57.7)			18,994 (53.2)		7658 (60.9)			64,420 (58.5)	
	One or more mobility, cognition, vision, or hearing disability	1459 (14)		43,046 (35.6)		5722 (34)			15,438 (43.2)		2985 (23.8)			38,965 (36.5)	
	Missing	4302 (41.4)		3960 (3.3)		1384 (8.2)			1292 (3.6)		1925 (3.6)			5323 (5)	
**Sex**															
	Male	4396 (42.3)		49,808 (41.2)		6957 (41.4)			13,662 (38.2)		5374 (42.8)			43,542 (40.8)	
	Female	5997 (57.7)		71,133 (58.8)		9850 (58.6)			22,061 (61.8)		7194 (57.2)			63,163 (59.2)	
	Missing	0 (0)		2 (0)		0 (0)			1 (0)		0 (0)			3 (0)	
**Insurance type**															
	Private	3065 (29.5)		89,723 (74.2)		10,846 (64.5)			24,959 (69.8)		7114 (56.6)			73,608 (68.9)	
	Public	387 (3.8)		20,399 (16.9)		2782 (16.6)			6794 (19)		1433 (11.3)			17,748 (15.9)	
	Uninsured	32 (0.3)		543 (0.4)		52 (0.3)			177 (0.5)		45 (0.4)			487 (0.5)	
	Missing	6909 (66.5)		10,278 (8.5)		3127 (18.6)			3794 (10.6)		3976 (31.6)			14,865 (13.9)	

**Table 3:** Encounter status as the numbers of phone, video, and in-person visits across time periods.

Encounter status		T1a (March 2019 to May 2019), n (%)		T1b (May 2019 to September 2019), n (%)		T2 (March 2020 to May 2020), n (%)	T3 (May 2020 to September 2020), n (%)
**In person**							
	Canceled		20,387 (17)		60,503 (17.6)	34,252 (32.6)	75,884 (20.6)
	Completed		47,900 (39.8)		138,209 (40.2)	7547 (7.2)	74,386 (20.2)
	Missed		3946 (3.3)		11,551 (3.3)	1701 (1.6)	7287 (2)
**Phone**							
	Canceled		1 (0)		5 (0)	44 (0)	44 (0)
	Completed		47,519 (39.5)		132,703 (38.6)	43,522 (41.4)	156,826 (42.6)
	Missed		480 (0.4)		1156 (0.3)	831 (0.8)	1246 (0.3)
**Video**							
	Canceled		0 (0)		1 (0)	266 (0.3)	1259 (0.3)
	Completed		17 (0)		21 (0)	16,309 (15.5)	50,241 (13.6)
	Missed		0 (0)		1 (0)	658 (0.6)	1437 (0.4)

**Table 4:** Main effects from the adjusted generalized estimating equation (GEE) for completed encounters interacted by time, controlled for patient sex and showing the odds of an encounter being completed given a particular patient characteristic.

Characteristic	T1^a^, OR^b^ (95% CI)	T2^c^, OR (95% CI)	T3^d^, OR (95% CI)
Non-Hispanic Black	0.77 (0.75-0.79)^e^*	1.32 (1.25-1.38)^e^*	1.07 (1.03-1.1)^e^*
Non-Hispanic Asian	1.01 (0.98-1.05)	0.79 (0.74-0.84)^e^*	0.97 (0.93-1)^e^**
Non-Hispanic other race	0.9 (0.86-0.95)^e^*	1.01 (0.92-1.1)	1.03 (0.97-1.08)
Hispanic	0.9 (0.84-0.95)^e^*	1.08 (0.97-1.2)	1.12 (1.04-1.19)^e^*
≥65 years old	1.24 (1.21-1.26)^e^*	0.81 (0.78-0.84)^e^*	0.92 (0.9-0.94)^e^*
One or more significant comorbidities	1.05 (1.03-1.07)^e^*	1.12 (1.08-1.16)^e^*	0.92 (0.9-0.94)^e^*
One or more mobility, cognition, vision, or hearing disability	1.04 (1.02-1.06)^e^*	1.15 (1.12-1.19)^e^*	1.12 (1.1-1.14)^e^*

^a^March 2019 to September 2019.

^b^OR: odds ratio.

^c^March 2020 to May 2020.

^d^May 2020 to September 2020.

^e^*P* values for the main effects; see Table S8 in Multimedia Appendix 1 for the interaction *P* values.

*Significant at *P*=.01.

**Significant at *P*=.10.

**Table 5:** Main effects from the adjusted generalized estimating equation (GEE) for canceled encounters interacted by time, controlled for patient sex and showing the odds of an encounter being completed given a particular patient characteristic.

Characteristic	T1^a^, OR^b^ (95% CI)	T2^c^, OR (95% CI)	T3^d^, OR (95% CI)
Non-Hispanic Black	0.97 (0.95-1)^e^*	0.64 (0.61-0.67)^e^**	0.74 (0.72-0.76)^e^**
Non-Hispanic Asian	1.02 (0.99-1.06)	1.28 (1.2-1.38)^e^**	1.08 (1.04-1.12)^e^**
Non-Hispanic other race	1.06 (1-1.11)^e^***	0.94 (0.86-1.03)	0.93 (0.88-0.98)^e^***
Hispanic	0.96 (0.9-1.02)	0.79 (0.71-0.89)^e^**	0.77 (0.71-0.83)^e^**
≥65 years old	0.9 (0.88-0.92)^e^**	1.31 (1.27-1.36)^e^**	1.15 (1.13-1.18)^e^**
One or more significant comorbidities	0.95 (0.93-0.97)^e^**	0.88 (0.85-0.91)^e^**	1.07 (1.05-1.09)^e^**
One or more mobility, cognition, vision, or hearing disability	0.97 (0.95-0.99)^e^**	0.86 (0.84-0.89)^e^**	0.88 (0.8-0.84)^e^**

^a^March 2019 to September 2019.

^b^OR: odds ratio.

^c^March 2020 to May 2020.

^d^May 2020 to September 2020.

^e^*P* values for the main effects; see Table S8 in Multimedia Appendix 1 for the interaction *P* values.

*Significant at *P*=.10.

**Significant at *P*=.01.

***Significant at *P*=.05.

**Table 6:** Main effects from the adjusted generalized estimating equation (GEE) for missed encounters interacted by time, controlled for patient sex and showing the odds of an encounter being completed given a particular patient characteristic.

Characteristic	T1^a^, OR^b^ (95% CI)	T2^c^, OR (95% CI)	T3^d^, OR (95% CI)
Non-Hispanic Black	2.87 (2.74-3)^e^*	2.27 (2.07-2.5)^e^*	2.63 (2.49-2.78)^e^*
Non-Hispanic Asian	0.86 (0.79-0.93)^e^*	0.98 (0.82-1.17)	0.8 (0.72-0.89)^e^*
Non-Hispanic other race	1.32 (1.18-1.47)^e^*	1.51 (1.23-1.84)^e^*	1.34 (1.19-1.52)^e^*
Hispanic	1.87 (1.68-2.09)^e^*	2.28 (1.86-2.81)^e^*	1.98 (1.74-2.25)^e^*
≥65 years old	0.47 (0.45-0.49)^e^*	0.6 (0.54-0.66)^e^*	0.57 (0.54-0.6)^e^*
One or more significant comorbidities	0.97 (0.93-1.02)	1.06 (0.97-1.15)	1.09 (1.04-1.15)^e^*
One or more mobility, cognition, vision, or hearing disability	0.93 (0.89-0.97)^e^*	0.97 (0.89-1.05)	0.99 (0.95-1.04)

^a^March 2019 to September 2019.

^b^OR: odds ratio.

^c^March 2020 to May 2020.

^d^May 2020 to September 2020.

^e^*P* values for the main effects; see Table S8 in Multimedia Appendix 1 for the interaction *P* values.

*Significant at *P*=.01.

**Table 7:** Main effects from the adjusted generalized estimating equation (GEE) for completed encounters during the COVID-19 pandemic (T2 and T3 only) interacted by visit modality, controlled for patient sex and encounter time period and showing the odds of an encounter being completed given a particular patient or encounter characteristic.

Characteristic	In-person, OR^a^ (95% CI)	Video, OR (95% CI)	Phone, OR (95% CI)
Non-Hispanic Black	1.07 (1.03-1.1)^b^*	0.63 (0.58-0.7)^b^*	0.79 (0.69-0.9)^b^*
Non-Hispanic Asian	1.04 (1-1.07)^b^**	1.02 (0.87-1.2)	0.81 (0.67-0.97)^b^***
Non-Hispanic other race	1 (0.94-1.06)	0.83 (0.68-1)^b^**	0.77 (0.6-0.99)^b^***
Hispanic	1.19 (1.1-1.28)^b^*	0.69 (0.56-0.86)^b^*	0.8 (0.58-1.08)
≥65 years old	0.86 (0.84-0.88)^b^*	1.08 (0.99-1.18)^b^**	0.96 (0.86-1.07)
One or more significant comorbidities	0.84 (0.83-0.86)^b^*	0.86 (0.8-0.93)^b^*	1 (0.9-1.11)
One or more mobility, cognition, vision, or hearing disability	1.07 (1.05-1.09)^b^*	0.91 (0.85-0.98)^b^***	1.09 (0.99-1.21)^b^**

^a^OR: odds ratio.

^b^*P* values for the main effects; see Table S9 in Multimedia Appendix 1 for the interaction *P* values.

*Significant at *P*=.01.

**Significant at *P*=.10.

***Significant at *P*=.05.

### Patient Characteristics Associated With Encounter Completion Status Over Time

#### Older Adults

Prior to the COVID-19 pandemic, among all scheduled encounters, older adults aged ≥65 years had greater odds of having completed encounters (adjusted OR [AOR] 1.24, 95% CI 1.21-1.26) and decreased odds of canceled visits (AOR 0.9, 95% CI 0.88-0.92), as compared with those <65 years of age; however, the opposite was true in the COVID-19 era. In the initial months of the COVID-19 pandemic (T2), scheduled encounters had lesser odds of being completed (AOR 0.81, 95% CI 0.78-0.84) and greater odds of being canceled (AOR 1.31, 95% CI 1.27-1.36) when patients were older adults, compared with when patients were <65 years of age. As the pandemic wore on into T3, the odds of encounter completion increased among appointments for older adults, and the odds of encounter cancellation decreased; however, returns to baseline were not achieved. Among scheduled encounters, the odds of encounter completion remained lower (AOR 0.92, 95% CI 0.9-0.94), and the odds of encounter cancellation were greater (AOR 1.17, 95% CI 1.15-1.2) than for appointments for adults <65 years of age. In addition, appointments for older adults ≥65 years old had the lowest odds of being no-shows at all time points compared with adults <65 years of age (T1: AOR 0.47, 95% CI 0.45-0.49; T2: AOR 0.6, 95% CI 0.54-0.66; T3: AOR 0.57, 95% CI 0.54-0.6). In terms of encounter modality, no significant differences were found between adults ≥65 years of age and those <65 years old regarding video and phone encounter completion during the COVID-19 shutdown; however, older adults’ in-person appointments were less likely to be completed compared with those for patients <65 years of age (AOR 0.84, 95% CI 0.84-0.88).

#### Comorbidities

Our data indicate that, prior to the COVID-19 pandemic, encounters for patients with at least one comorbidity were more likely to be completed than encounters for those without comorbidities (AOR 1.05, 95% CI 1.03-1.07). During the initial days of the pandemic (T2), our data show those with at least one comorbidity had even greater odds of completing encounters (AOR 1.12, 95% CI 1.08-1.16); however, they had lower odds of completing encounters as the pandemic continued (T3: AOR 0.92, 95% CI 0.9-0.94). Conversely, encounters for those patients with comorbidities were less likely to be canceled than encounters for those without comorbidities in our historical control prior to the COVID-19 pandemic (AOR 0.95, 95% CI 0.93-0.97), and this remained true during T2 in the early days of the pandemic (AOR 0.88, 95% CI 0.85-0.91). However, as the pandemic lingered, encounters for patients with comorbidities had greater odds of cancellation compared with those without comorbidities (AOR 1.09, 95% CI 1.06-1.11).

#### Disability (Cognitive, Mobility, Vision, or Hearing)

Our models included both comorbidities and disability status as measures. These were intended to represent different concerns around equity of telehealth and were utilized despite some conceptual overlap between the 2 variables. However, modeling was done with both variables, as well as with each variable separately. The inclusion of both did not impact the significance nor direction of effect for either comorbidities or disabilities.

Results showed that, in all 3 time periods, encounters for patients with at least one disability were more likely to be completed than encounters for those without disabilities, with a slight increase in effect size from the pre-COVID-19 time period (AOR 1.04, 95% CI 1.02-1.06) to the 2 COVID-19 time periods (T2: AOR 1.15, 95% CI 1.12-1.19; T3: AOR 1.12, 95% CI 1.1-1.14). Conversely, encounters for these patients were significantly less likely to be canceled in all 3 time periods, with a slightly smaller effect in T1 (AOR 0.97, 95% CI 0.95-0.99) relative to T2 (AOR 0.86, 95% CI 0.84-0.89) and T3 (AOR 0.88, 95% CI 0.87-0.9). Patient disabilities had no significant effect on missed encounter odds during the COVID-19 time periods but were associated with slightly reduced odds of missed encounters in the pre-COVID-19 time period (AOR 0.93, 95% CI 0.89-0.97). In terms of visit modality, encounters for patients with at least one disability were significantly more likely to be completed when they were in-person encounters (AOR 1.07, 95% CI 1.05-1.09), as compared with those without disabilities; however, the opposite was true for video encounters (AOR 0.91, 95% CI 0.85-0.98). Thus, there was a significant 9% reduction in odds that a video visit would be completed than was the case for patients without disabilities.

#### Black Patients

Data from our study suggest that the COVID-19 pandemic also had an impact on odds of encounter completion among Black patients. Prior to the pandemic, scheduled encounters for non-Hispanic Black patients were less likely to be completed than those for non-Hispanic White patients (AOR 0.77, 95% CI 0.75-0.79), whereas during the COVID-19 time periods (T2 and T3), encounters for non-Hispanic Black patients were more likely to be completed and were less likely to be canceled (T2: AOR 0.64, 95% CI 0.61-0.67; T3: AOR 0.74, 95% CI 0.72-0.76). Odds of encounters in which patients did not show were higher when patients were non-Hispanic Black across all 3 time periods; however, there was an initial reduction in the size of this effect at T2 (AOR 2.27, 95% CI 2.07-2.5) followed by a return toward the control effect size (AOR 2.87, 95% CI 2.74-3.0) by T3 (AOR 2.63, 95% CI 2.49-2.78). Regarding visit modality, Black patients were more likely to complete in-person encounters compared with non-Hispanic White patients (AOR 1.07, 95% CI 1.03-1.1) but less likely to complete encounters via video (AOR 0.63, 95% CI 0.58-0.7) or phone (AOR 0.79, 95% CI 0.69-0.9) during the COVID-19 shutdown.

#### Hispanic Patients

Data indicate that, prior to the pandemic, encounter completion was less likely when patients were Hispanic than when patients were non-Hispanic White (AOR 0.9, 95% CI 0.84-0.95). During the COVID-19 pandemic, however, appointments for these patients were more likely to be completed during T3 (AOR 1.12, 95% CI 1.04-1.19). In addition, although encounters for Hispanic patients were less likely to be canceled during the pandemic (T2: AOR 0.79, 95% CI 0.71-0.89; T3: AOR 0.77, 95% CI 0.71-0.83), they remained much more likely to have missed encounters throughout the full study period, as compared with encounters for non-Hispanic White patients. This had an increasing effect size between T1 (AOR 1.87, 95% CI 1.68-2.09) and T2 (AOR 2.28, 95% CI 1.85-2.81), only to return to close to control by T3 (AOR 1.98, 95% CI 1.74-2.25). Regarding visit modality, Hispanic patients were more likely to complete in-person encounters compared with non-Hispanic White patients (AOR 1.19, 95% CI 1.1-1.28) but less likely to complete encounters via video (AOR 0.69, 95% CI 0.56-0.86).

#### Asian Patients

In this study, although their encounters were not significantly more or less likely to be completed than those for White patients in our historical control, Asian patients’ encounters were less likely to be completed when scheduled during the early COVID-19 period (T2: AOR 0.79, 95% CI 0.74-0.84). Encounters for Asian patients were also the only subset found to be more likely to be canceled during both T2 (AOR 1.28, 95% CI 1.2-1.36) and T3 (AOR 1.07, 95% CI 1.03-1.11). Compared with White patients, Asian patients were less likely to miss encounters prior to the pandemic (AOR 0.86, 95% CI 0.79-0.93) and later in the pandemic (T3: AOR 0.8, 95% CI 0.72-0.89). In terms of modality, no significant differences were found between White patients and Asian patients for in-person or video encounter completion, but Asian patients were found to be less likely to complete phone-based encounters than White patients (AOR 0.81, 95% CI 0.67-0.97).

## Discussion

### Principal Findings

Findings from this study demonstrate that primary care utilization across delivery modalities (in person, video, and phone) was not equivalent across all groups before and during the COVID-19 pandemic, and different groups were differentially impacted at different time points. Patterns of patient groups who completed, canceled, or missed their appointments across these modalities also differed. Although video and phone-based visits were increased to try to maintain access to care during the pandemic, patterns of utilization of different modalities suggest that COVID-19 and changes in how care was delivered affected different patient populations in different ways.

### Overall Encounter Status

Analysis indicates that the odds of completing health care encounters dramatically changed in our health care system during the early stages of the pandemic, which is consistent with the work of others [19,20]. Compared with a comparable historical control from March 16, 2019, to September 30, 2019, we saw a 56% reduction in completed in-person encounters and an 11% increase in phone-based care in 2020. Of particular interest is the rapid and dramatic expansion of video-based telehealth usage within our system during the pandemic, in which nearly 66,500 primary care encounters were completed compared with 38 in a comparable time period in 2019. This major expansion is consistent with other work that has documented the incredible telehealth expansion seen across the United States in the early stages of the pandemic [21-23].

### Effect of the COVID-19 Pandemic on Health Care Provision by Groups

#### Older Adults

Overall, findings from this study suggest that the COVID-19 pandemic adversely affected older adults with regard to completing and canceling scheduled encounters. Reasons for these dramatic shifts in encounter completion and cancellation patterns are likely attributed to the fact that, since the pandemic began, older adults have been at higher risk for greater COVID-19 disease severity, including hospitalization and death [24]. Because of this increased COVID-19–related morbidity and mortality, not only were older adults likely self-selecting to avoid in-person encounters early in the pandemic but health care systems also preemptively canceled appointments and changed visit modalities in an effort to limit patient exposure to COVID-19. Indeed, about one-third of older adults have reported avoiding urgent, emergency, and routine medical care early in the pandemic [25]. Of the approximately one-third of older adults who delayed care, nearly one-quarter reported, 6 months to 10 months after the start of the COVID-19 pandemic, that their care had not been completed, and only about one-half reported that some of their delayed care had been completed. Moreover, of those who had delayed care, nearly 20% reported that these delays had adversely affected their health [25,26]. Results from the National Healthy Poll on Aging suggested that vaccine status may influence a patient’s likelihood of catching up on missed care, as vaccinated or vaccinated and boosted survey respondents were more likely to have rescheduled disrupted care in January 2022 than unvaccinated respondents [27].

#### Comorbidities

Data from our study suggest that patients with comorbidities received more care during the initial days of the pandemic and less as the pandemic continued. Explanations for these patterns may be attributed to possible prioritization of those patients who could least afford to delay or forego care during this time, coupled with the fact that patients with certain comorbidities were more likely to experience greater COVID-19 disease severity, with increased hospitalizations and death, necessitating timely health care [28,29]. Surprisingly though, these patterns did not continue during T3, during which, in fact, we found that patients with comorbidities were less likely to have completed encounters and were more likely to have canceled encounters. Reasons for these findings are unclear but could possibly be because they had been seen already during T2, or perhaps this is reflective of the health system bringing healthier patients back in during T3 due to delays in care. It was also interesting to note that patients with comorbidities were less likely to complete in-person visits, which may be explained by increased risk of COVID-19–related morbidity and mortality in this group; however, patients with comorbidities were also less likely to complete video encounters, which may reflect a patient preference for in-person visits or potential increased need to actually be seen in person for things that could not be assessed virtually (eg, physical assessments, need for lab draws).

#### Disability (Cognitive, Mobility, Vision, or Hearing)

Our findings show that people with disabilities were more likely to complete and less likely to cancel encounters across all time periods, compared with people without disabilities. Potential explanations for these findings include the idea that patients with disabilities may have had greater need for care, which is supported by the fact that people living with disabilities have poorer overall health [30,31]. Furthermore, although not all individuals with disabilities are at increased risk for contracting or experiencing severe COVID-19, some subgroups, including those with cognitive disabilities, may experience COVID-19–related health inequities [32], which may also partially explain our findings. Our investigation also showed that those with disabilities were more likely to complete visits in person but less likely to complete video visits. The decreased odds of completing video visits may potentially speak to technology access, technology literacy issues, and health literacy issues. For example, Americans with disabilities are less likely to own different kinds of digital devices [33], which may prevent access to virtual care. Additionally, those who are deaf or hard of hearing have lower rates of health literacy [34,35] and worse access to technology, especially for older adults [33,36,37], than those without hearing impairment [38]. Furthermore, technologies used for telehealth may not be accessible to people with visual disabilities due to visual navigation or print size. People with physical disabilities may also need assistance with certain types of examinations, making in-person visits necessary [38].

#### Black Patients

Our data demonstrate that the COVID-19 pandemic increased the odds of encounter completion and decreased the odds of encounter cancellation among Black patients. Reasons for these shifts are unclear; however, these findings could be partially attributed to the fact that the spread of COVID-19 was affecting communities of color worse, both in terms of prevalence and severity [10]. Reasons for these health inequities are likely nuanced and multifactorial and are likely tied to social determinants of health [39-42], including higher rates of chronic disease and comorbid conditions; nature of work, including high rates of frontline workers; access to health care; increased reliance on public transportation; living in more densely populated communities and homes; and structural racism. Specific to this study, our work was conducted within Michigan Medicine, the health system affiliated with the University of Michigan, located in Ann Arbor, MI. Ann Arbor is considered by many to be an outlying suburb of Detroit, as it is located fewer than 50 miles from the Detroit city center. Detroit is a city that is about 80% Black and was a major epicenter for early COVID-19 waves. Given Ann Arbor’s proximity to Detroit, COVID-19’s impact on the Black community was profoundly felt in our health system.

In our study, despite the increased odds of encounter completion and decreased odds of encounter cancellation, we found that Black patients were less likely to use telehealth than non-Hispanic White patients. This is a finding that is consistent with other findings in the literature [43,44]. Inequity in telehealth adoption among Black patients is likely complex and nuanced. In a recent investigation of telehealth use within a federally qualified health center network in Texas, Black patients were less likely to utilize telehealth [43], suggesting that the intersection of socioeconomic factors and factors related to social determinants of health likely play important roles in health and health care inequities.

Lower telehealth utilization among Black patients may also be attributed to the manner through which telehealth is often accessed. In many hospital systems, telehealth access is tied to patient portal use, which may be problematic. Significant inequities have been shown to exist, with Black and Hispanic patients being less likely than White patients to be offered patient portal access and, among those offered, are also less likely to use patient portals [45]. Improving inequities in access and use of patient portals may be one step toward improving inequities in telehealth utilization.

#### Hispanic Patients

Similar to our findings concerning Black patients, our data indicate that encounters were less likely to be completed and more likely to be canceled by Hispanic patients than by non-Hispanic White patients prior to the COVID-19 pandemic. However, they were more likely to be completed during T3 of the pandemic and less likely to be canceled in both pandemic time periods. This contrasts with related work that showed Hispanic patients were less likely to complete video visits during the pandemic [46]. Moreover, Hispanic patients had higher rates of missed appointments in all time periods compared with White patients. As in our findings concerning Black patients, these findings are likely attributable to greater COVID-19 morbidity and mortality among Hispanic populations, coupled with increased health inequities and adverse social determinants of health experienced by Hispanic patients compared with non-Hispanic White patients. Indeed, compared with non-Hispanic White patients, Hispanic patients have had higher ratios of COVID-19 infections, hospitalizations, and death [47]. When looking at age-adjusted infection rates over time, Hispanic patients have been disproportionately affected by COVID-19 compared with White patients, particularly early in the pandemic [47-50]. In addition, as with Black patients, reasons for this inequity have been attributed to disproportionate representation of Hispanic patients in frontline and essential worker job roles [51], as well as other social determinants. Additionally, our results suggest that Hispanic patients were more likely to complete encounters in person compared with non-Hispanic White patients and less likely to complete video visits. For Spanish-speaking patients with limited English proficiency, this may be linked to early difficulties at the study site in incorporating interpreters into video visits. Indeed, the study site changed video visit platform vendors in T3 partly due to this barrier. Moreover, findings from our related work revealed that a Spanish language version of our patient portal was not available when the pandemic began, and even when this version was rolled out, it was limited in functionality [38].

#### Asian Patients

Results from this investigation indicate that, compared with White patients, Asian patients were less likely to complete encounters during the early days of the pandemic and more likely to cancel encounters during both COVID-19 time periods. These findings are particularly interesting to note, as at first glance, it may appear that Asian Americans have not experienced COVID-19–related inequities in terms of prevalence as compared with other groups who have been historically marginalized [10]; however, emerging evidence suggests that the COVID-19–related burden on this population may be greater than realized and the true prevalence rate of COVID-19 in this population may be drastically underestimated [52]. Reasons for this underestimation are thought to include lack of testing [53], possibly due to potential concerns of racism [52]. Indeed, anti-Asian xenophobia has been well-documented during the COVID-19 era [54-56], and the potential impact this may have on health inequities has been acknowledged [57,58]. For example, our finding that Asian patients were less likely to complete and more likely to cancel encounters may be potentially attributed to possible fear of xenophobic interactions. Differential impacts on Asian Americans have been largely ignored in the mainstream media, but emerging perspectives are scrutinizing the impact of the COVID-19 pandemic on this population more fully [59]. However, alternative explanations for these patterns of completing and canceling encounters could also be attributable in the early days of the pandemic to the fact that there may have been increased sensitivity to avoiding the virus among Asian patients given that the global outbreak originated in Asia. Our findings also demonstrated that Asian patients were less likely to complete encounters via phone than White patients, which may be attributed to cultural beliefs: Related work from our team has suggested that some of our Asian patients believe that healing is associated with being with a physician in person [38].

### Limitations

This study is not without limitations. Our data set was composed of elements extracted from our EHR, which is not always easily interpretable. Indeed, EHR data are not compiled for research purposes, and secondary data analyses should be interpreted with some degree of caution, as EHR data are often plagued with data quality issues such as incompleteness, inconsistencies, and inaccuracies [60-62]. As a result of the limitations of secondary analysis of our EHR data, we were not able to assess certain characteristics at the patient level, such as insurance status. Moreover, this study only explored adult primary care encounters and did not include urgent care nor emergency care visits, which potentially represent important clinical encounter types during the pandemic; it also did not include pediatric appointments. Future work should focus on understanding whether the COVID-19 pandemic had differential effects on the utilization of video and audio telehealth visits among other primary care settings such as pediatrics, as well as for patients seeking care in urgent care and emergency department settings. Additionally, although we took multiple steps to identify which phone visits were actual health care visits and exclude other, nonvisit phone calls, there still may be some phone encounters in our data set that were not scheduled health care visits but rather were some other interactions with the health system.

One major limitation of our approach is the fact that we were only able to investigate primary care clinical encounters that were scheduled within our health system. Given the concerning findings from the Centers for Disease Control and Prevention suggesting an estimated 41% of US adults either avoided or delayed medical care during the first few months of the pandemic [63], we were not able to comment on actual access-to-care issues experienced by patients in our health system and instead could only look at utilization. More robust work is needed to better understand the effect of COVID-19 on delayed and missed care, which will likely have ripple effects for decades to come. Further, we cannot infer reasons for canceled encounters, although recent work suggests that provider cancellations and fear of contracting COVID-19 may be large drivers [64]. Although we can surmise that many patients were likely to have deliberately delayed or avoided care during the early stages of the pandemic, we also had no way of capturing the number of patients who may have been turned away for care due to lack of access to in-person appointment slots, lack of availability of video or phone-based appointments, or lack of access to suitable technology to complete video or phone-based encounters. Moreover, as the only patients represented in our data set were those who had an actual scheduled encounter, our completion models for each time period, which compare patients with completed encounters with patients who canceled or missed their appointments, may be skewed. These limitations suggest a need for future work to more fully explain the effects of the COVID-19 pandemic on access to, and utilization of, care across multiple modalities. This work will likely require more focused effort, utilizing more in-depth study designs. Our research group has been exploring some of these factors from both the patient and provider perspectives, using mixed method approaches, which will contribute to this growing body of knowledge.

### Conclusion

In summary, the COVID-19 pandemic ushered in a seismic shift in how care was delivered to patients, with video and phone-based care rocketing to the forefront of our solutions to extend access to care. However, it is clear that this rapid expansion of care modalities was met with differential rates of utilization among different communities of patients. Understanding how different groups of patients responded to these rapid changes and how health care inequities may have been affected is an important step in better understanding implementation strategies for digital solutions in the future. Clearly, more emphasis needs to be paid to how implementations of digital health solutions affect different subpopulations of patients in order to refrain from exacerbating inequities among groups of patients. It is hoped that, with more careful attention paid, we may be better able to reduce, as opposed to exacerbate, inequity.

## Appendix

Multimedia Appendix 1 Supplementary tables.

## Abbreviations

AOR: adjusted odds ratio
EHR: electronic health record
GEE: generalized estimating equation
ICD: International Classification of Diseases
OR: odds ratio
STROBE: Strengthening the Reporting of Observational Studies in Epidemiology

## Data Availability

The participants of this study did not give written consent for their data to be shared publicly. As these are HIPAA-protected data, sharing of the research-supporting data is not available.

## References

[1] Dellifraine JL, Dansky KH (2008). Home-based telehealth: a review and meta-analysis. J Telemed Telecare.

[2] Dorsey ER, Topol EJ (2016). State of telehealth. N Engl J Med.

[3] Grigsby J, Rigby M, Hiemstra A, House M, Olsson S, Whitten P (2002). Telemedicine/telehealth: an international perspective. The diffusion of telemedicine. Telemed J E Health.

[4] Whitten PS, Mackert MS (2005). Addressing telehealth's foremost barrier: Provider as initial gatekeeper. Int J Technol Assess Health Care.

[5] Ye S, Kronish I, Fleck E, Fleischut P, Homma S, Masini D, Moise N (2021). Telemedicine expansion during the COVID-19 pandemic and the potential for technology-driven disparities. J Gen Intern Med.

[6] Meyer PA, Yoon PW, Kaufmann RB, Centers for Disease Control and Prevention (CDC) (2013). Introduction: CDC Health Disparities and Inequalities Report - United States, 2013. MMWR Suppl.

[7] Abrams EM, Szefler SJ (2020). COVID-19 and the impact of social determinants of health. The Lancet Respiratory Medicine.

[8] Backer S, Rezene A, Kahar P, Khanna D (2022). Socioeconomic determinants of COVID-19 incidence and mortality in Florida. Cureus.

[9] Factors That Affect Your Risk of Getting Very Sick from COVID-19. Centers for Disease Control and Prevention.

[10] Ndugga N, Hill L, Artiga S (2022). COVID-19 Cases and Deaths, Vaccinations, and Treatments by Race/Ethnicity as of Fall 2022. KFF.

[11] Shahid Z, Kalayanamitra R, McClafferty B, Kepko D, Ramgobin D, Patel R, Aggarwal CS, Vunnam R, Sahu N, Bhatt D, Jones K, Golamari R, Jain R (2020). COVID-19 and older adults: what we know. J Am Geriatr Soc.

[12] Anderson M (2019). Mobile Technology and Home Broadband 2019. Pew Research Center.

[13] Elston Lafata J, Miller CA, Shires DA, Dyer K, Ratliff SM, Schreiber M (2018). Patients' adoption of and feature access within electronic patient portals. Am J Manag Care.

[14] Veinot T, Mitchell H, Ancker JS (2018). Good intentions are not enough: how informatics interventions can worsen inequality. J Am Med Inform Assoc.

[15] (2021). The R Project for Statistical Computing. R Project.

[16] von Elm E, Altman DG, Egger M, Pocock SJ, Gøtzsche PC, Vandenbroucke JP (2007). The Strengthening the Reporting of Observational Studies in Epidemiology (STROBE) statement: guidelines for reporting observational studies. The Lancet.

[17] Charlson ME, Pompei P, Ales KL, MacKenzie C (1987). A new method of classifying prognostic comorbidity in longitudinal studies: development and validation. J Chronic Dis.

[18] Gasparini A (2018). comorbidity: An R package for computing comorbidity scores. Journal of Open Source Software.

[19] Mehrotra A, Chernew ME, Linetsky D, Hatch H, Cutler DM, Schneider EC (2021). The Impact of COVID-19 on Outpatient Visits in 2020: Visits Remained Stable, Despite a Late Surge in Cases. The Commonwealth Fund.

[20] Whaley CM, Pera MF, Cantor J, Chang J, Velasco J, Hagg HK, Sood N, Bravata DM (2020). Changes in health services use among commercially insured US populations during the COVID-19 pandemic. JAMA Netw Open.

[21] Demeke HB, Merali S, Marks S, Pao LZ, Romero L, Sandhu P, Clark H, Clara A, McDow KB, Tindall E, Campbell S, Bolton J, Le X, Skapik JL, Nwaise I, Rose MA, Strona FV, Nelson C, Siza C (2021). Trends in use of telehealth among health centers during the COVID-19 pandemic - United States, June 26-November 6, 2020. MMWR Morb Mortal Wkly Rep.

[22] Friedman AB, Gervasi S, Song H, Bond AM, Chen AT, Bergman A, David G, Bailey JM, Brooks R, Smith-McLallen A (2022). Telemedicine catches on: changes in the utilization of telemedicine services during the COVID-19 pandemic. Am J Manag Care.

[23] Samson LK, Tarazi W, Turrini G, Sheingold S (2021). Medicare Beneficiaries’ Use of Telehealth in 2020: Trends by Beneficiary Characteristics and Location. Assistant Secretary for Planning and Evaluation.

[24] COVID-19 Risks and Information for Older Adults. Centers for Disease Control and Prevention.

[25] Malani P (2021). One-third of older Americans delayed health care over COVID concerns. National Poll on Healthy Aging.

[26] Zhong S, Huisingh-Scheetz M, Huang ES (2022). Delayed medical care and its perceived health impact among US older adults during the COVID-19 pandemic. J Am Geriatr Soc.

[27] Kullgren J, Malani P (2022). Pandemic disruptions mean many older adults still haven’t gotten needed care. National Poll on Healthy Aging.

[28] (2023). Underlying Medical Conditions Associated with Higher Risk for Severe COVID-19: Information for Healthcare Professionals. Centers for Disease Control and Prevention.

[29] Sanyaolu A, Okorie C, Marinkovic A, Patidar R, Younis K, Desai P, Hosein Z, Padda I, Mangat J, Altaf M (2020). Comorbidity and its impact on patients with COVID-19. SN Compr Clin Med.

[30] Havercamp SM, Scott HM (2015). National health surveillance of adults with disabilities, adults with intellectual and developmental disabilities, and adults with no disabilities. Disabil Health J.

[31] Mitra M, Long-Bellil L, Moura I, Miles A, Kaye HS (2022). Advancing health equity and reducing health disparities for people with disabilities in the United States. Health Aff (Millwood).

[32] Turk MA, Landes SD, Formica MK, Goss KD (2020). Intellectual and developmental disability and COVID-19 case-fatality trends: TriNetX analysis. Disabil Health J.

[33] Perrin A, Atske S (2021). Americans with disabilities less likely than those without to own some digital devices. Pew Research Center.

[34] McKee MM, Paasche-Orlow MK, Winters PC, Fiscella K, Zazove P, Sen A, Pearson T (2015). Assessing health literacy in deaf American sign language users. J Health Commun.

[35] Wells TS, Rush S, Musich S, Nickels L, Wu L, Yeh CS (2018). Impacts of hearing loss and limited health literacy among older adults.

[36] Valentine G, Skelton T, Levy P, Maruyama T (2019). The Role of the Internet in D/Deaf People's Inclusion in the Information Society. SlidePlayer.

[37] Valentine G, Skelton T (2009). ‘An umbilical cord to the world’. Information, Communication & Society.

[38] Guetterman Timothy C, Koptyra Emily, Ritchie Olivia, Marquis Liz B, Kadri Reema, Laurie Anna, Vydiswaran Vg Vinod, Li Jiazhao, Brown Lindsay K, Veinot Tiffany C, Buis Lorraine R (2023). Equity in virtual care: A mixed methods study of perspectives from physicians. J Telemed Telecare.

[39] Robertson MM, Shamsunder MG, Brazier E, Mantravadi M, Zimba R, Rane MS, Westmoreland DA, Parcesepe AM, Maroko AR, Kulkarni SG, Grov C, Nash D (2022). Racial/ethnic disparities in exposure, disease susceptibility, and clinical outcomes during COVID-19 pandemic in national cohort of adults, United States. Emerg Infect Dis.

[40] Vasquez Reyes M (2020). The disproportional impact of COVID-19 on African Americans. Health Hum Rights.

[41] Wachtler B, Michalski N, Nowossadeck E, Diercke M, Wahrendorf M, Santos-Hövener C, Lampert T, Hoebel J (2020). Socioeconomic inequalities and COVID-19 - A review of the current international literature. J Health Monit.

[42] Webb Hooper M, Nápoles AM, Pérez-Stable EJ (2020). COVID-19 and racial/ethnic disparities. JAMA.

[43] Adepoju OE, Chae M, Ojinnaka CO, Shetty S, Angelocci T (2022). Utilization gaps during the COVID-19 pandemic: racial and ethnic disparities in telemedicine uptake in Federally Qualified Health Center Clinics. J Gen Intern Med.

[44] Karimi M, Lee EC, Couture SJ, Gonzales A, Grigorescu V, Smith SR, De Lew N, Sommers BD (2022). National Survey Trends in Telehealth Use in 2021: Disparities in Utilization and Audio vs. Video Services. Assistant Secretary for Planning and Evaluation.

[45] Richwine C, Johnson C, Patel V (2023). Disparities in patient portal access and the role of providers in encouraging access and use. J Am Med Inform Assoc.

[46] Rodriguez JA, Betancourt JR, Sequist TD, Ganguli I (2021). Differences in the use of telephone and video telemedicine visits during the COVID-19 pandemic. Am J Manag Care.

[47] National Center for Immunization and Respiratory Diseases (2021). Risk for COVID-19 infection, hospitalization, and death by race/ethnicity. Centers for Disease Control and Prevention.

[48] Gold JA, Rossen LM, Ahmad FB, Sutton P, Li Z, Salvatore PP, Coyle JP, DeCuir J, Baack BN, Durant TM, Dominguez KL, Henley SJ, Annor FB, Fuld J, Dee DL, Bhattarai A, Jackson BR (2020). Race, ethnicity, and age trends in persons who died from COVID-19 - United States, May-August 2020. MMWR Morb Mortal Wkly Rep.

[49] Ndugga N, Hill S, Artiga S (2022). COVID-19 Cases and Deaths, Vaccinations, and Treatments by Race/Ethnicity as of Fall 2022. KFF.

[50] Romano SD, Blackstock AJ, Taylor EV, El Burai Felix S, Adjei S, Singleton C, Fuld J, Bruce BB, Boehmer TK (2021). Trends in racial and ethnic disparities in COVID-19 hospitalizations, by region - United States, March-December 2020. MMWR Morb Mortal Wkly Rep.

[51] Do DP, Frank R (2021). Using race- and age-specific COVID-19 case data to investigate the determinants of the excess COVID-19 mortality burden among Hispanic Americans. DemRes.

[52] Yan BW, Hwang AL, Ng F, Chu JN, Tsoh JY, Nguyen TT (2021). Death toll of COVID-19 on Asian Americans: disparities revealed. J Gen Intern Med.

[53] Rubin-Miller L, Alban C, Artiga S, Sullivan S (2020). COVID-19 Racial Disparities in Testing, Infection, Hospitalization, and Death: Analysis of Epic Patient Data. KFF.

[54] Findling MG, Blendon RJ, Benson J, Koh H (2022). COVID-19 Has Driven Racism And Violence Against Asian Americans: Perspectives From 12 National Polls. HealthAffairs.

[55] Gray C, Hansen K (2021). Did Covid-19 lead to an increase in hate crimes toward Chinese people in London?. Journal of Contemporary Criminal Justice.

[56] Tessler H, Choi M, Kao G (2020). The anxiety of being Asian American: hate crimes and negative biases during the COVID-19 pandemic. Am J Crim Justice.

[57] Le TK, Cha L, Han H, Tseng W (2020). Anti-Asian xenophobia and Asian American COVID-19 disparities. Am J Public Health.

[58] Santos PMG, Dee EC, Deville C (2021). Confronting anti-Asian racism and health disparities in the era of COVID-19. JAMA Health Forum.

[59] Yee A (2021). COVID’s Outsize Impact on Asian Americans Is Being Ignored. Scientific American.

[60] Botsis T, Hartvigsen G, Chen F, Weng C (2010). Secondary use of EHR: data quality issues and informatics opportunities. Summit Transl Bioinform.

[61] Edmondson M, Reimer AP (2020). Challenges frequently encountered in the secondary use of electronic medical record data for research. Comput Inform Nurs.

[62] Sarwar T, Seifollahi S, Chan J, Zhang X, Aksakalli V, Hudson I, Verspoor K, Cavedon L (2022). The secondary use of electronic health records for data mining: data characteristics and challenges. ACM Comput. Surv.

[63] Czeisler MÉ, Marynak K, Clarke KE, Salah Z, Shakya I, Thierry JM, Ali N, McMillan H, Wiley JF, Weaver MD, Czeisler CA, Rajaratnam SM, Howard ME (2020). Delay or avoidance of medical care because of COVID-19-related concerns - United States, June 2020. MMWR Morb Mortal Wkly Rep.

[64] Ni B, Gettler E, Stern R, Munro HM, Steinwandel M, Aldrich MC, Friedman DL, Sanderson M, Schlundt D, Aronoff DM, Gupta DK, Shrubsole MJ, Lipworth L (2021). Disruption of medical care among individuals in the southeastern United States during the COVID-19 pandemic. J Public Health Res.

[65] Javerbaum M, Houghton M (2020). Google supports COVID-19 AI and data analytics projects. The Keyword: Google.

