# Supplementary material for: Identifying Inequities in Video and Audio Telehealth Services for Primary Care Encounters During COVID-19: Repeated Cross-Sectional, Observational Study
Source: J Med Internet Res. 2023 Sep 29;25:e49804. doi: 10.2196/49804 (PMC10544805; doi:10.2196/49804)
Supplement: Multimedia Appendix 1 [file jmir_v25i1e49804_app1.docx]

Appendix tables

| Table S8. Interaction P-Values for Encounter Status by Time GEE Models (see *Tables 4-6*) | | | | | | | | |  | |  | |  | |
| --- | --- | --- | --- | --- | --- | --- | --- | --- | --- | --- | --- | --- | --- | --- |
|  | **Completed Encounter** | | | **Cancelled Encounter** | | | | | | **No Show Encounter** | | | | |
|  | **T1 vs T2** | **T2 vs T3** | **T1 vs T3** | | **T1 vs T2** | **T2 vs T3** | **T1 vs T3** | **T1 vs T2** | | | | **T2 vs T3** | | **T1 vs T3** |
| NH Black | *<0.001* | *<0.001* | *<0.001* | | *<0.001* | *<0.001* | *<0.001* | *<0.001* | | | | *0.008* | | *0.017* |
| NH Asian | *<0.001* | *<0.001* | *0.028* | | *<0.001* | *<0.001* | *0.028* | 0.197 | | | | *0.054* | | 0.280 |
| NH Other Race | *0.042* | 0.705 | *<0.001* | | *0.032* | 0.836 | *<0.001* | 0.242 | | | | 0.340 | | 0.789 |
| Hispanic | *0.004* | 0.550 | *<0.001* | | *0.007* | 0.527 | *<0.001* | *0.086* | | | | 0.240 | | 0.500 |
| 65+ yrs | *<0.001* | *<0.001* | *<0.001* | | *<0.001* | *<0.001* | *<0.001* | *<0.001* | | | | 0.298 | | *<0.001* |
| One or more significant comorbidities | *0.001* | *<0.001* | *<0.001* | | *<0.001* | *<0.001* | *<0.001* | *0.090* | | | | 0.529 | | *<0.001* |
| One or more mobility, cognition, vision, and/or hearing disability | *<0.001* | *0.095* | *<0.001* | | *<0.001* | 0.196 | *<0.001* | 0.336 | | | | 0.602 | | *0.022* |

| Table S9. Interaction P-Values for Encounter Completion by Visit Modality GEE Model (see *Table 7*) | | | |
| --- | --- | --- | --- |
|  | **In Person vs Video Visit** | **Phone vs Video Visit** | **In Person vs Phone Visit** |
| NH Black | *<0.001* | *0.007* | *<0.001* |
| NH Asian | 0.973 | *0.044* | *0.009* |
| NH Other Race | *0.059* | 0.711 | *0.053* |
| Hispanic | *<0.001* | 0.494 | *0.014* |
| ≥65 | *<0.001* | *0.058* | 0.103 |
| One or more significant comorbidities | 0.577 | *0.025* | *0.002* |
| One or more mobility, cognition, vision, and/or hearing disability | *<0.001* | *0.004* | 0.691 |
